# Beyond lifestyle modification: the role of GLP-1 receptor agonists in treating pediatric obesity

**DOI:** 10.3389/fped.2025.1684485

**Published:** 2026-01-20

**Authors:** Hua Wen

**Affiliations:** Department of Pediatrics, Hospital of Chengdu University of Traditional Chinese Medicine, Chengdu, China

**Keywords:** adolescents, clinical applications, GLP-1 receptor agonists (GLP-1 RAs), pediatric obesity, weight loss

## Abstract

Childhood obesity is a global health crisis with limited effective therapies beyond lifestyle modification. This review examines the growing role of glucagon-like peptide-1 receptor agonists (GLP-1 RAs) in the management of pediatric obesity. Simultaneously, this study highlights the critical considerations for clinicians in administering these medications, including long-term safety, efficacy, potential adverse effects, and overall clinical utility. To enhance the effectiveness of GLP-1 RAs in managing pediatric obesity, we propose a comprehensive clinical medication use management process that includes review, screening, combination therapy, education, monitoring, and follow-up. We also present recommendations for policymakers and healthcare systems. In conclusion, GLP-1 RAs represent an emerging therapeutic paradigm for pediatric obesity, demonstrating notable efficacy in weight reduction for adolescents. Ongoing research is needed to determine long-term outcomes, optimal treatment duration, and strategies to ensure broad and equitable access to these therapies.

## 1 Introduction

Childhood obesity has risen dramatically worldwide. Overweight and obesity in youths (5–19 years) climbed from roughly 8% in 1990 to about 20% by 2022, affecting approximately 160 million young people globally. In the United States, current data indicate that about 20%–22% of adolescents (ages 12–19) have obesity (1). This epidemic carries serious health consequences. Pediatric obesity is strongly linked to early-onset type 2 diabetes, hypertension, dyslipidemia, nonalcoholic fatty liver, and orthopedic problems, as well as psychosocial issues such as poor self-esteem and depression (2). Moreover, excess weight in childhood tends to persist: youth with obesity are several-fold more likely to become adults with obesity (3), compounding lifetime risks of cardiovascular disease and metabolic disorders. These trends make pediatric obesity a pressing public-health crisis demanding more effective treatments.

Currently, the primary intervention for childhood obesity is standard care (healthy diet, exercise, and behavior counseling), pharmacotherapy, and weight loss surgery (1, 4). A preponderance of evidence suggests that routine assessments and counseling interventions are somewhat effective for preventing and treating obesity in children (1, 5–7). But the efficacy varies widely among patients, likely depending on the timing and intensity of the intervention, patient age, and family economics, as well as genetic or other fixed factors that contribute to obesity (1). Due to drug approval, regulatory, and age-related reasons, the medication options for treating pediatric obesity are very limited, and clinicians are only advised to prescribe them to children with obesity over 12 years of age. Phentermine is only approved for short-term use (12 weeks) in adolescents older than 16 years of age (8). Weight loss surgery (gastric bypass or sleeve gastrectomy) reliably induces large, durable weight loss and remission of comorbidities (9). However, surgery is invasive, costly, and accessible only at specialized centers; it carries operative risks and long-term nutritional concerns in growing youths. Surgery is typically reserved for the most severe cases after failure of lifestyle therapy. In sum, there has been a significant “treatment gap” between the massive population of youth with obesity and the small number of safe, effective non-surgical interventions.

Glucagon-like peptide-1 receptor agonists (GLP-1 RAs) have recently offered new hope. GLP-1 is an incretin hormone that enhances insulin secretion, suppresses glucagon, slows gastric emptying, and acts on the hypothalamus to promote satiety (10). Pharmacologic GLP-1 RAs (such as liraglutide and semaglutide) exploit these effects to reduce appetite and caloric intake, leading to weight loss while improving glycemic control. They were first developed for type 2 diabetes in adults, where striking weight loss was observed as a side effect. Given their efficacy and acceptable safety in adults, GLP-1 RAs became logical candidates for trials in adolescents with obesity, a group with few other medication options.

Since 2020, multiple randomized trials have confirmed the significant benefits of GLP-1 RAs in adolescents, which can induce substantial, clinically meaningful weight loss and metabolic benefits in adolescents with obesity. Moreover, the weight loss achieved with GLP-1 RAs combined with lifestyle intervention approaches is comparable to that of surgical intervention, far surpassing the results adolescents can achieve through lifestyle changes alone (11–13). Even with strong evidence confirming the efficacy of GLP-1 RAs in the field of pediatric obesity, there are still many issues that warrant attention. For example: adverse drug reactions, long-term safety, optimal timing of treatment, weight maintenance after discontinuation, effects on children's growth and development, and the cost of drug access.

This paper is a narrative review that references the SANRA guidelines for narrative reviews, but was not strictly written according to these guidelines. To prepare this review, we searched several databases, including PubMed, Embase, and Web of Science. Time Window (Start and End Dates): The search included studies published from the database inception to July 2025. Inclusion/Exclusion Criteria: Studies were included based on the following criteria: (1) study type: randomized controlled trial; (2) population: children and adolescents under 18 years of age; (3) intervention: GLP-1 receptor agonists; (4) outcomes: relevant to pediatric and adolescent obesity. Studies were excluded if they were not high-quality clinical trials or did not focus on the use of GLP-1 receptor agonists in pediatric and adolescent populations. We specifically excluded studies that did not investigate the use of GLP-1 receptor agonists for weight loss in children and adolescents. Study Selection Process: The study selection process involved screening titles and abstracts of articles identified through the database searches. Duplicates were identified and removed. Full-text articles of potentially relevant studies were then retrieved and assessed for eligibility based on the inclusion and exclusion criteria. Quality/Risk-of-Bias Assessment: No formal quality or risk-of-bias assessment was performed. This decision was made because this review aims to provide a general overview of the current literature landscape and summarize the progress of research in this emerging area, rather than to conduct a formal meta-analysis or systematic review. We focused on selecting high-quality clinical trials to ensure the reliability of the included studies.

This article synthesizes high-quality clinical evidence to provide a comprehensive review of the mechanisms and therapeutic advantages of GLP-1 RAs in the treatment of pediatric obesity. The focus is on key considerations for clinicians when selecting these medications, including long-term safety, efficacy, potential adverse effects, and overall clinical utility. The synthesis aims to serve as a valuable reference for clinicians, policymakers, and decision-makers within healthcare systems. To optimize the effectiveness of GLP-1 RAs in addressing pediatric obesity, we propose a comprehensive management process for medication use. This process encompasses a detailed review of the patient's medical history, thorough screening for contraindications and potential drug interactions, consideration of combination therapies to enhance efficacy, comprehensive education for both patients and their families, rigorous monitoring of treatment response and side effects, and structured follow-up appointments to ensure long-term management and adherence. Future research directions in the utilization of GLP-1 RAs for pediatric obesity treatment center on several key areas. These include the investigation of novel agents and optimal dosing strategies, assessment of long-term outcomes and safety profiles, evaluation of psychosocial impacts and strategies to promote health equity, and optimization of real-world implementation through combination therapies and digital health tools. A critical emphasis is placed on continuous monitoring for adverse effects and ensuring the long-term efficacy and safety of these agents in the pediatric population, with the ultimate goal of defining optimal treatment regimens and identifying patient populations that derive maximal benefit.

### 1.1 GLP-1 RAs: mechanisms and current medications

GLP-1, an incretin hormone secreted by intestinal L-cells, plays a crucial role in postprandial metabolic homeostasis (14). Physiologically, GLP-1 enhances glucose-stimulated insulin secretion and suppresses glucagon release (14, 15). It also reduces caloric intake by slowing gastric emptying and activating central satiety circuits in the brainstem and hypothalamic nuclei (16). Studies in rodent models have demonstrated that central GLP-1 signaling induces satiety independently of gastric signals. These multifaceted actions make GLP-1 an attractive target for obesity treatment (14, 17, 18).

However, native GLP-1 is rapidly degraded by dipeptidyl peptidase-4 (DPP-4) (19). Pharmaceutical GLP-1 receptor agonists (GLP-1RAs) have been engineered to resist DPP-4 degradation, resulting in prolonged circulating stability (20, 21). Current GLP-1RAs include human GLP-1 analogs (e.g., liraglutide, dulaglutide, semaglutide) and exendin-4–derived peptides (e.g., exenatide, lixisenatide), each modified to extend half-life through various mechanisms such as amino acid substitution and lipidation (14). Clinically, GLP-1RAs offer the advantage of acting only in the presence of hyperglycemia, minimizing hypoglycemia risk. While various GLP-1RAs have demonstrated efficacy in promoting weight loss, clinicians should be aware of the differences in their pharmacokinetic profiles and potential side effects when selecting an appropriate agent for individual patients (20). Further research is needed to fully elucidate the long-term effects and comparative effectiveness of different GLP-1RAs in adolescent obesity.

### 1.2 GLP-1 RAs and weight loss

GLP-1 RAs exert weight-reduction effects by both slowing gut transit and directly activating anorexigenic neural circuits. The anorectic mechanism of GLP-1 RAs derives from their ability to mimic endogenous GLP-1's gut-brain signaling. In patients with obesity, GLP-1 RA therapy blunts the rise in ghrelin and other orexigenic gut peptides, thereby suppressing hunger (22, 23).

As a result of these actions, GLP-1 RAs produce clinically meaningful weight loss. Meta-analyses and trials in adults have consistently shown that GLP-1 RA therapy lowers body weight by ∼5%–15% depending on agent and dose (17, 24). In type 2 diabetes trials, for example, liraglutide at 3.0 mg daily induced ∼5%–7% weight loss over one year, and semaglutide 2.4 mg weekly produced ∼10%–15% weight loss in non-diabetic patients with obesity (25–27). Multiple systematic reviews and meta-analyses focused on pediatric populations have demonstrated that GLP-1 receptor agonists (GLP-1 RAs) significantly reduce blood pressure and HbA1c levels while promoting weight loss. These effects are further amplified when GLP-1 RAs are combined with lifestyle interventions. Moreover, the associated side effects are relatively minimal, primarily presenting as gastrointestinal (GI) discomfort (28–30). Weight loss on GLP-1 RAs is accompanied by improvements in cardiometabolic markers (glycemia, lipids, blood pressure) and has spurred investigation of GLP-1 RA therapy for obesity itself, independent of diabetes.

Clinically, several GLP-1 RAs are approved or under study for obesity. Liraglutide 3.0 mg once daily (brand Saxenda) and semaglutide 2.4 mg once weekly (brand Wegovy) have been explicitly FDA-approved for chronic weight management. Both agents mimic endogenous GLP-1 but differ in pharmacokinetics: liraglutide requires daily injection, whereas semaglutide's extended half-life allows weekly dosing. These approvals are grounded in pediatric and adult trials (11, 12, 31, 32). Overall, current guidelines recognize liraglutide and semaglutide as first-line pharmacotherapy options for severe adolescent obesity when combined with lifestyle therapy.

Notwithstanding the rapid evolution of pharmacotherapeutic options, for pediatric and adolescent patients with obesity, standard care (healthy diet, exercise, and behavior counseling)—defined as the integration of healthy dietary adjustments, regular physical activity, and behavioral counseling—remains the first-line treatment. Drug-based and surgical approaches should be considered solely as adjunctive measures supplementing this fundamental approach (1). The selection of treatment strategies for pediatric and adolescent obesity necessitates a patient-centered approach. Clinicians should comprehensively evaluate factors such as patient age, weight, comorbidities, prior treatments, and family circumstances to formulate the optimal therapeutic plan.

When lifestyle interventions prove insufficient, pharmacotherapy and metabolic/bariatric surgery (MBS) merit consideration. Glucagon-like peptide-1 receptor agonists (GLP-1 RAs), such as liraglutide and semaglutide, are receiving increased attention in the management of severe adolescent obesity due to initial signals of efficacy. However, before widespread adoption, a measured assessment of their differentiation from existing modalities is warranted. In clinical practice, GLP-1 RAs are not a universal solution. Over-reliance on pharmacotherapy without adequate commitment to lifestyle interventions may obfuscate underlying etiologies. Phentermine/topiramate, while offering potential benefit as an adjunct, is limited by its adverse effect profile. Metabolic/bariatric surgery, despite its marked efficacy, remains a last-line option due to its invasiveness and potential for complications (Table 1). Therefore, in the context of adolescent obesity, a paradigm emphasizing “lifestyle intervention as the cornerstone, with pharmacotherapy and surgery as adjuncts” should be upheld, avoiding inversion of fundamental principles. Therapeutic decision-making necessitates comprehensive consideration of individual patient characteristics, meticulous evaluation of the advantages and disadvantages of each modality, and transparent communication with patients and their families to collaboratively formulate the most appropriate treatment plan.

**Table 1:** Concise comparison of treatment modalities for obesity in children and adolescents.

Feature	GLP-1 RAs (liraglutide, semaglutide)	Phentermine/topiramate	MBS (metabolic/bariatric surgery)
Efficacy	Moderate to High: While initial data are promising, long-term outcomes require further substantiation, with notable inter-individual variability observed.	Moderate: As an adjunct, efficacy is circumscribed, exhibiting substantial dependence on concomitant lifestyle modifications.	High: Currently, the most efficacious modality, yet the inherent invasiveness and potential sequelae necessitate careful deliberation.
Durability	Variable, contingent on adherence: Adherence represents a critical determinant; cessation of pharmacotherapy may precipitate weight regain.	Limited: Insufficient data exist regarding the long-term safety and sustained efficacy profiles.	Potentially Long-Term: Provided patients maintain adherence to a healthful lifestyle, effects may be sustained over extended durations.
Risks	Gastrointestinal adverse events, pancreatitis risk: Common adverse events may impact quality of life; the infrequent, albeit serious, risk of pancreatitis warrants vigilance.	Psychiatric effects, cardiovascular risk: Potential psychiatric sequelae constrain widespread application; cardiovascular risks necessitate close monitoring.	Surgical complications, nutritional deficiencies: Surgical interventions inherently pose risks; long-term management of potential nutritional deficiencies is essential.
Indications	Severe obesity (BMI ≥ 95th percentile) unresponsive to lifestyle interventions: May be considered following the failure of lifestyle modification, contingent upon meticulous risk-benefit assessment.	Adjunctive treatment for severe obesity: Typically not a first-line approach, reserved for select cases as a supplementary measure.	Class 2/3 obesity with comorbidities or Class 3 obesity: Reserved for severe cases refractory to other interventions, representing a last-resort strategy.
Pediatric Evidence	Accumulating: Pediatric evidence is growing, yet additional longitudinal data are required to validate safety and efficacy parameters.	Limited: Data pertaining to pediatric populations remain circumscribed, necessitating caution.	Established: Ample pediatric research supports utilization, albeit with stringent adherence to established indications.

### 1.3 GLP-1 RAs: clinical benefits and current applications

Robust RCTs have demonstrated striking weight loss with GLP-1 RAs in adolescents (Table 2). In the 68-week Phase 3 STEP TEENS trial, weekly semaglutide (2.4 mg) plus lifestyle produced a mean −16.1% change in BMI from baseline vs. +0.6% with placebo. In practical terms, 76% of semaglutide-treated participants experienced a BMI reduction of ≥5% (vs. 23% on placebo) and 63% experienced a BMI reduction of ≥10% (vs. 10% on placebo) (12). Similarly, a 56-week trial of daily liraglutide (3.0 mg) in 12–17-year-olds showed significantly greater BMI reduction than lifestyle alone. About 43.3% of liraglutide-treated adolescents experienced a ≥5% reduction in BMI (compared to 18.7% in the placebo group), and 26.1% of liraglutide-treated adolescents experienced a ≥10% reduction in BMI (compared to 8.1% in the placebo group). Notably, weight tended to rebound after treatment stopped—for example, BMI-SDS rose again when liraglutide was withdrawn– indicating that ongoing therapy may be required to sustain benefit (11). In a 68-week clinical trial of 200 adolescents with obesity, weekly administration of 2.4 mg semaglutide resulted in significant improvements in BMI classification. Specifically, 44.9% of participants achieved a BMI consistent with normal weight or overweight status, compared to 12.1% in the placebo group. Importantly, these benefits were observed across all subgroups analyzed, irrespective of gender or age (33).

**Table 2:** Clinical application studies of GLP-1 RAs in pediatric obesity.

Study	Age group	Number randomized	Study duration	Difference, GLP-1 RAs vs. Placebo (95% CI)	Adverse events	Other Observed Outcomes, GLP-1 RAs vs. Placebo (95% CI)
Kelly AS et al. (11)	12 to <18	Liraglutide 3.0 mg	56 weeks	BMI (%): −4.64	Any adverse events: 88.8% (Placebo 84.9%);	≥5% BMI loss: 43.3% vs. 18.7%; ≥10% BMI loss: 26.1% vs. 8.1%;
		(*N* = 125)		(−4.29 vs. 0.35);	Gastrointestinal disorders: 64.8% (Placebo 36.5%).	≥5% body weight loss and ≥10% body weight loss: N/A.
		Placebo		Body Weight (kg): −4.5		Glycemic and cardiometabolic variables or in overall weight-related quality of life: no substantial difference.
		(*N* = 126)		(−2.26 vs. 2.25).		
Weghuber D et al. (12)	12 to <18	Semaglutide 2.4 mg	68 weeks	BMI (%): −16.7 (−16.1 vs. 0.6)	Any adverse events: 79% (Placebo 82%);	≥5% body weight loss: 73% vs. 18%; ≥10% body weight loss: 62% vs. 8%;
		(*N* = 132)		Body Weight (kg): −17.7	Gastrointestinal disorders: 62% (Placebo 42%).	≥5% BMI loss: 76% vs. 23%; ≥10% BMI loss: 63% vs. 10%.;
		Placebo		(−15.3 vs. 2.4)		Waist circumference (cm): −12.1 (−12.7 vs. −0.6);
		(*N* = 67)				Glycated hemoglobin (%): −0.3 (−0.4 vs. −0.1);
						Total cholesterol (%): −7.1 (−8.3 vs. −1.3);
						Low-density lipoprotein cholesterol (%): −7.0 (−10.2 vs. −3.4);
						Very-low-density lipoprotein cholesterol (%): −29.5 (−28.4 vs. 1.6);
						Triglycerides (%): −30.3 (−28.4 vs. 2.6);
						ALT (%): −14.1 (−18.3 vs. −4.9).
Kelly AS et al. (33)	12 to <18	Semaglutide 2.4 mg	68 weeks	An improvement of at least one BMI category: 54.7% (73.7% vs. 19%)	N/A	N/A
		(*N* = 133)		An improvement of at least two BMI categories: 41.5% (44.9% vs. 3.4%)		
		Placebo				
		(*N* = 67)				
Fox CK et al. (35)	6 to <12	Liraglutide 3.0 mg	56 weeks	BMI (%): −7.4 (−5.8 vs. 1.6);	Any adverse events: 89%	≥5% BMI loss: 46% vs. 9%;
		(*N* = 56)		Body Weight (kg): −6.0	(Placebo 88%);	≥10% BMI loss: 35% vs. 4%;
		Placebo		(1.1 vs. 7.1).	Gastrointestinal disorders: 80% (Placebo 54%).	≥5% body weight loss and ≥10% body weight loss: N/A.
		(*N* = 26)				Systolic blood pressure (%): −3.4 (−1.7 vs. 1.7);
						Diastolic blood pressure (mm Hg): −4.2 (−1.2 vs. 3);
						Glycated hemoglobin (%): −0.1 (−0.2 vs. −0.1).

In addition to weight loss, GLP-1 RAs improve metabolic health in youth with obesity. In the semaglutide trial, treated adolescents showed significantly larger declines in glycated hemoglobin (HbA1c), fasting glucose, waist circumference, triglycerides, LDL-cholesterol and alanine aminotransferase than placebo (10). Another study demonstrated that semaglutide improves several cardiometabolic risk factors (ALT, LDL cholesterol, triglycerides, waist circumference, systolic blood pressure, and diastolic blood pressure), except glycated hemoglobin (HbA1c) (33). A recent meta-analysis of 14 RCTs in adolescents with overweight or obesity (including those with type 2 diabetes) confirmed that GLP-1 RA therapy led to mean HbA1c reductions (−0.34% risk difference) and weight loss (−4.3 kg) relative to the control group (34). Smaller pediatric studies have also noted improvements in insulin sensitivity (e.g., lower fasting insulin or HOMA-IR) and even blood pressure with GLP-1 RA–induced weight loss. For example, in a 56-week trial of liraglutide in 6–12-year-olds, the drug group experienced greater reductions in systolic blood pressure and blood glucose than the placebo (35). These data suggest that cardiometabolic risk factors improve in parallel with weight. (Quality-of-life measures generally improve as obesity and its symptoms remit, although formal QoL endpoints have been reported only in a limited fashion).

Despite dramatic short-term results, many questions remain. To date, almost all high-quality pediatric clinical trials have been relatively brief (typically 1–2 years) (11, 12, 34, 36), so the long-term efficacy and safety of GLP-1 RAs in youth are unknown. It is unclear how long treatment must continue to maintain weight loss, and whether chronic therapy is needed (as in adults)—especially since drug cessation leads to rapid weight regain (11). Crucially, the evidence for children under 12 years is preliminary and limited, with currently only one large clinical trial available. A recent 56-week trial in 6–12-year-olds showed BMI reductions with liraglutide, but investigators cautioned that long-term effects on growth, puberty and development are undetermined (35). Likewise, minority and low-income youths have been underrepresented in trials, so we lack evidence on GLP-1 RA effects across diverse racial/ethnic groups. Finally, studies have rarely examined non-weight outcomes (e.g., final adult height, bone density, neurocognitive or psychosocial changes) systematically. Ongoing studies—including open-label extensions and trials in younger children—are needed to address these gaps and guide best use of GLP-1 therapy in pediatric obesity.

Even though clinical studies show significant weight loss with GLP-1 RAs, and their use in youth has surged, access and application remain uneven. In the U.S., adolescent prescriptions for FDA-approved obesity medications (primarily high-dose semaglutide and liraglutide) rose by roughly 300% from 2020 to 2023, following new approvals and guideline endorsements. Nonetheless, only about 0.5% of teens with obesity received any such medication in 2023. Prescribing is concentrated in older teens, girls, and those with more severe obesity: for example, adolescents with class 2 or 3 obesity had 4–13 times higher prescription rates than those with milder obesity. By contrast, Black adolescents were significantly less likely than White adolescents to receive anti-obesity medications (adjusted prevalence ratio ∼0.61) (36). These patterns highlight persistent inequities: although effective drugs exist, high cost and limited insurance coverage mean that only a small, advantaged subset of youths benefit.

### 1.4 Safety, tolerability, and practical considerations

GLP-1 RAs, such as liraglutide and semaglutide, are increasingly used as adjuncts to diet/exercise in pediatric obesity. In clinical trials in children and adolescents, these agents have been generally well tolerated, with gastrointestinal symptoms by far the most common adverse events. Meta-analyses and large trials report high rates of nausea, vomiting, diarrhea, and constipation in treated youths, typically mild to moderate in severity and diminishing over time (29, 37). For example, in a 56-week trial of liraglutide in children aged 6–11 years with obesity, 89% of treated children reported an adverse event (mainly GI) vs. 88% on placebo, with 80% of liraglutide subjects experiencing GI symptoms (e.g., nausea, vomiting, or diarrhea) compared to 54% of placebo. In another trial of liraglutide treatment for adolescents aged 12–17 years, 88.8% of treated adolescents experienced adverse events, with 64.8% reporting nausea, vomiting, or diarrhea (compared to 36.5% of adolescents in the placebo group, where 84.9% experienced adverse events) (11, 35). Likewise, once weekly semaglutide in adolescents caused GI effects in 62% of patients vs. 42% on placebo (12). These side effects were generally mild-to-moderate and often transient. Other common side effects include transient headache, fatigue or dizziness, and injection-site reactions, but significant hypoglycemia is rare when GLP-1 RAs are used alone (hypoglycemia generally only occurs if combined with insulin or sulfonylureas) (15). Current data indicate that these medications do not appear to cause growth retardation or affect pubertal progression in the short term; however, there is a lack of long-term data to substantiate this claim (37, 38). Withdrawals due to side effects are uncommon and comparable to placebo, indicating overall good tolerability (29, 35).

While generally safe, GLP-1 RAs carry a few serious but uncommon risks that clinicians must be aware of and monitor. In the treatment of pediatric obesity, GLP-1 therapy may be associated with gallbladder diseases, particularly cholelithiasis. In the 2022 semaglutide trial in teens, 5 of 133 (≈4%) treated subjects developed gallstones vs. none on placebo (12). Liraglutide and semaglutide carry labeling for “acute gallbladder disease” and pancreatitis (15), and one adolescent treated with liraglutide experienced a single episode of pancreatitis, which was of moderate severity and resolved without treatment. An increase in amylase and lipase levels from baseline to week 68 was observed with semaglutide, but no cases of pancreatitis were reported (11, 12). GLP-1 RAs also raise heart rate slightly (a few beats per minute on average) and may worsen dehydration in susceptible patients (since vomiting/diarrhoea can precipitate acute kidney injury) (37). A boxed warning exists for medullary thyroid carcinoma risk (based on rodent data), so use is contraindicated in familial MEN2 or personal thyroid C-cell cancer history. Of note, one adolescent in the liraglutide group died by suicide during the trial, and one adolescent reported a suicide attempt (deemed unrelated to the study drug) (11). This underscores the importance for clinicians to conduct psychological health screenings with caution in practice, especially considering that obesity itself is associated with social and psychological risks. In practice, careful screening for depression is prudent, especially since obesity itself carries psychosocial risk. No increase in malignancies or microvascular complications has been observed in youth, but trials have been too short to detect infrequent events. Overall, the side-effect profile in children mirrors that in adults: predominantly GI, mostly transient, and rarely serious (12, 29).

Long-term safety outlook for pediatric use remains cautiously optimistic but uncertain. Currently available pediatric trials span roughly one year or less, and extension studies are ongoing. There is no evidence of accelerated growth suppression or unexpected toxicity so far, but prolonged exposure during growth has not been studied. Experts emphasize that GLP-1 RAs should be prescribed with close follow-up. If sufficient weight loss is not achieved (e.g., <4% BMI reduction at 12 weeks), therapy should be reconsidered (39). Drug discontinuation and rebound weight gain are possible if the medication is stopped. Because weight recidivism is common after drug treatment, indefinite or repeated courses may be needed, raising questions about cumulative long-term risks. In adults, registries have not shown major safety signals; however, pediatric obesity exhibits a different physiology. Thus, professional reviewers highlight the need for ongoing surveillance of bone health, neurodevelopment, and metabolic effects over years (37, 38). In summary, while current evidence suggests GLP-1 RAs are safe in the short-to-midterm, the “long game” in growing children is uncharted and warrants caution. In clinical practice, providers typically conduct a trial period (usually 12–16 weeks) to assess the effectiveness of the therapy, recommending cessation if weight loss is insufficient (40).

In practice, additional factors influence GLP-1 use. Cost and insurance coverage are major barriers: these drugs are expensive, branded biologics, and many insurers impose strict criteria (e.g., documented failure of lifestyle therapy, presence of comorbidities, high BMI thresholds) before approval. Even then, copayments or caps may limit continuous use. A recent review notes that lack of coverage and high out-of-pocket costs can lead to “treatment interruptions or discontinuation,” disproportionately affecting lower-income families (1). Meanwhile, effective weight management requires integration with behavioral therapy. All studies combine GLP-1 RAs with nutrition and exercise programs; standalone drug therapy is not recommended. In practice, providers reinforce dietitian-led counselling (e.g., high-protein, fibre-rich, frequent small meals) and physical activity to bolster weight loss and mitigate GI side effects (1, 41). Behavioral support (addressing eating behaviors, emotional triggers, psychological wellbeing) remains crucial for adherence and long-term success. In sum, GLP-1 RAs are viewed as powerful tools but not panaceas; they must be embedded in a comprehensive, family-centered treatment plan.

## 2 Discussion

The evidence to date indicates that GLP-1 RAs have significant potential for treating adolescent obesity, but careful integration into clinical practice is essential. Large randomized trials consistently show robust weight-loss efficacy. For example, semaglutide 2.4 mg weekly achieved dramatic BMI reduction in teens with severe obesity. In the STEP TEENS trial, nearly three-quarters of participants improved their BMI category by at least one level, and over 40% reached a BMI below the obesity threshold (33). Similarly, liraglutide 3.0 mg daily produced significantly greater BMI drops than placebo (35). These results far exceed the modest weight changes typically seen with lifestyle intervention alone. Importantly, weight loss with GLP-1 RAs has accompanied improvements in metabolic parameters and obesity-related comorbidities (e.g., glycemic control, lipid profiles, liver enzymes) in adolescents, mirroring findings in adults (see e.g., NASPGHAN and Endocrine guidelines).

Safety data in youth are generally reassuring but incomplete. Most adverse effects are gastrointestinal (nausea, vomiting, diarrhea) and tend to lessen with time or dose adjustment (37). In trials, serious adverse events are uncommon and did not differ significantly from placebo (35). Nonetheless, clinicians should be vigilant. FDA prescribing information (for approved agents) warns of the theoretical risk of thyroid C-cell tumors (based on animal data). It contraindicates use in patients with personal or family history of medullary thyroid carcinoma. Monitoring should also include kidney function, since dehydration from GI side effects could precipitate renal issues (37). Notably, no deterioration of bone mineral density has been seen; some studies suggest GLP-1 RAs may even have bone-preserving effects (37). However, given the rapid weight change and potential nutritional impact, it is prudent to monitor linear growth and puberty (height, bone age, sexual maturation) in any pediatric patient on these drugs (38, 42). Growth data from the long-term liraglutide or semaglutide studies in children will be especially informative. Clinicians should also screen for eating disorders or mood changes, since the psychosocial effects (positive or negative) of pharmacologic weight loss in youth are not yet fully understood (43).

Current clinical guidelines reflect this evolving evidence. In 2023 the American Academy of Pediatrics (AAP) and other expert bodies began endorsing earlier consideration of pharmacotherapy: weight-loss medications are now recommended for youth aged ≥12 (and even younger in controlled settings) who have obesity or overweight with comorbidities, after insufficient response to intensive lifestyle therapy. GLP-1 RAs are highlighted as first-line options given their efficacy. However, guidelines uniformly emphasize that medication must be used alongside continued diet and exercise counseling. Importantly, GLP-1 RAs should be prescribed and managed by clinicians experienced in pediatric obesity (e.g., pediatric endocrinologists or obesity specialists) because of dosing complexities and the need for growth monitoring. Physicians should also adopt a sensitive, patient-centered approach: explain that these are tools to improve health (not a “quick fix”), set realistic expectations, and address weight stigma.

Despite the promise of GLP-1 RAs, several evidence gaps and challenges temper full enthusiasm for these therapies. Long-term maintenance of weight loss after stopping therapy is a concern; the STEP TEENS trial showed weight rebound after discontinuation (as in adults), suggesting that many patients may need extended or intermittent treatment. The optimal duration of therapy and strategies for preventing weight regain are unknown. Additionally, most pediatric trials have been relatively short (1 year) and involve selected volunteers. Real-world adherence, especially in teenagers, may be lower. Insurance coverage is also a barrier: as Abass et al. note, many insurers impose strict criteria and high copays, limiting access (37). This could widen health disparities, since adolescents from under-resourced families already have lower access to specialty obesity care (43). Policymakers and payors therefore face a choice: support broader coverage of proven therapies (potentially offsetting long-term costs of obesity complications) or maintain restrictive policies.

In evaluating the application of GLP-1 RAs for the treatment of obesity in children, it is essential to consider the limitations of the existing evidence, as these limitations may significantly impact the reliability and generalizability of research findings. Firstly, the relatively short approval period of GLP-1 RAs in the pediatric obesity domain has resulted in a limited number of high-quality clinical studies. The lack of long-term, systematic research may prevent the current evidence from adequately reflecting the efficacy and safety of these drugs in a broader pediatric population. Additionally, due to individual variability and differences in developmental stages, the response to the medication may differ from that observed in adults, making it difficult to extrapolate the results of short-term studies to long-term usage scenarios. Secondly, there is a lack of uniformity in the efficacy evaluation methods employed across studies. For instance, the use of different assessment metrics such as Body Mass Index (BMI), BMI standard deviation score (BMI-SDS), BMI loss, and BMI categories contributes to the complexity of result comparisons. This lack of standardization diminishes the comparability of results and hinders an accurate assessment of the efficacy of GLP-1 RAs. Moreover, existing research has shown relative deficiencies in the collection of data concerning side effects and potential clinical risks. Many studies have failed to comprehensively evaluate all possible adverse effects, leading to uncertainties for clinicians when treating pediatric obesity, thereby impacting clinical decision-making. Finally, the duration of current pediatric clinical studies typically ranges from 1 to 2 years, which is insufficient to provide robust support for the long-term safety of GLP-1 RAs. Short follow-up periods may not capture potential safety issues associated with long-term use, thereby increasing the unknown risks to the long-term health of pediatric patients.

### 2.1 Given these gaps, we propose the following recommendations based on current evidence

#### 2.1.1 For clinicians

We propose a clinical medication use management process that encompasses review, screening, combination therapy, education, monitoring, and follow-up (Table 3).

**Table 3:** Recommendations for clinicians: clinical medication management process.

Step	Content
Review	- Conduct a rigorous assessment of medication indications. For eligible children with obesity, it is advisable to initiate treatment as early as possible.
Screening	- Perform comprehensive screening and evaluation prior to the commencement of pharmacotherapy. This assessment should include: - Evaluation of thyroid function and familial history of thyroid disorders, - Screening for gallbladder diseases, - Assessment of pancreatic conditions, - Evaluation of renal function, - Psychological health assessment.
Combination	- Pharmacotherapy must always be integrated with lifestyle interventions to optimize treatment outcomes.
Education	- Educate families regarding potential adverse effects (e.g., gastrointestinal symptoms), the importance of meal adherence to mitigate nausea, as well as the recognition of signs indicative of gallbladder or pancreatic concerns.
Monitoring	- Continuously monitor the parameters evaluated during screening. Assess weight reduction dynamically and establish a trial period of 12 to 16 weeks—discontinuing the medication if no significant weight loss is observed. - Monitor growth and developmental parameters, including bone age, pubertal progression, variations in growth metrics, and psychological health status. - Track relevant laboratory indices associated with treatment.
Follow-Up	- Following the discontinuation of medication, maintain oversight of weight management outcomes, prompt the development of maintenance strategies, and contribute data to inform long-term outcomes.

The specific details are as follows:

##### 2.1.1.1 Review

- Conduct a rigorous assessment of medication indications. For eligible children with obesity, it is advisable to initiate treatment as early as possible.

##### 2.1.1.2 Screening

- Perform comprehensive screening and evaluation prior to the commencement of pharmacotherapy.
This assessment should include:
- Evaluation of thyroid function and familial history of thyroid disorders,
- Screening for gallbladder diseases,
- Assessment of pancreatic conditions,
- Evaluation of renal function,
- Psychological health assessment.

##### 2.1.1.3 Combination

- Pharmacotherapy must always be integrated with lifestyle interventions to optimize treatment outcomes.

##### 2.1.1.4 Education

- Educate families regarding potential adverse effects (e.g., gastrointestinal symptoms), the importance of meal adherence to mitigate nausea, as well as the recognition of signs indicative of gallbladder or pancreatic concerns.

##### 2.1.1.5 Monitoring

- Continuously monitor the parameters evaluated during screening. Assess weight reduction dynamically and establish a trial period of 12–16 weeks—discontinuing the medication if no significant weight loss is observed.
- Monitor growth and developmental parameters, including bone age, pubertal progression, variations in growth metrics, and psychological health status.
- Track relevant laboratory indices associated with treatment.

##### 2.1.1.6 Follow-Up

- Following the discontinuation of medication, maintain oversight of weight management outcomes, prompt the development of maintenance strategies, and contribute data to inform long-term outcomes.

#### 2.1.2 For policymakers and healthcare systems

##### 2.1.2.1 Social advocacy

- Recognize pediatric obesity as a chronic condition necessitating comprehensive management. Support initiatives within educational institutions and community settings aimed at destigmatizing obesity and informing families about available medical treatments.

##### 2.1.2.2 Regulatory approvals

- Expedite the review process for pediatric indications of novel anti-obesity pharmacotherapies.

##### 2.1.2.3 Insurance coverage

- Ensure that insurance plans provide coverage for GLP-1 RAs for adolescents who meet the established clinical criteria, thereby minimizing bureaucratic barriers to access.

##### 2.1.2.4 Financial assistance

- Implement subsidies or patient assistance programs to address the substantial costs associated with treatment, thereby promoting equity in access to care.

##### 2.1.2.5 Research funding

- Allocate funding for comparative-effectiveness research on various treatment modalities for pediatric obesity, including assessments of GLP-1 RAs in relation to bariatric surgery.

##### 2.1.2.6 Research into optimal Use

- Advocate for research initiatives that focus on the optimal use of obesity treatments, including clinical trials designed to evaluate the efficacy of lower dosages or combination therapies (e.g., GLP-1 RAs in conjunction with metformin) specifically for adolescents.

Overall, while GLP-1 RAs are a breakthrough for adolescent obesity, judicious use is key. Clinicians must weigh benefits against unknowns (especially long-term) and engage patients in shared decision-making. Policymakers should help close access gaps so that all youth who need treatment can receive it. With these steps (Tables 3 and 4), the potential of GLP-1 RAs to improve teen health can be maximized while mitigating risks.

**Table 4:** Recommendations for policymakers and healthcare systems.

Recommendations	Content
Social Advocacy	- Recognize pediatric obesity as a chronic condition necessitating comprehensive management. Support initiatives within educational institutions and community settings aimed at destigmatizing obesity and informing families about available medical treatments.
Regulatory Approvals	- Expedite the review process for pediatric indications of novel anti-obesity pharmacotherapies.
Insurance Coverage	- Ensure that insurance plans provide coverage for GLP-1 receptor agonists (RAs) for adolescents who meet the established clinical criteria, thereby minimizing bureaucratic barriers to access.
Financial Assistance	- Implement subsidies or patient assistance programs to address the substantial costs associated with treatment, thereby promoting equity in access to care.
Research Funding	- Allocate funding for comparative-effectiveness research on various treatment modalities for pediatric obesity, including assessments of GLP-1 receptor agonists in relation to bariatric surgery.
Research into Optimal Use	- Advocate for research initiatives that focus on the optimal use of obesity treatments, including clinical trials designed to evaluate the efficacy of lower dosages or combination therapies (e.g., GLP-1 receptor agonists in conjunction with metformin) specifically for adolescents.

## 3 Future direction

Current and forthcoming research on GLP-1 RAs in pediatric obesity will focus on new agents, optimal dosing regimens, long-term outcomes, and comprehensive safety monitoring. Several trials are underway investigating dual- and triple-incretin agonists. For example, tirzepatide—a dual GLP-1/GIP agonist already approved for adult obesity—is being evaluated in adolescents with obesity. Novel tri-agonists (targeting GLP-1, GIP, and glucagon) like retatrutide are in early development (37, 44) Adult data show that tirzepatide produces substantially greater BMI reduction than GLP-1 monotherapy (37), suggesting these agents could revolutionize pediatric treatment if found safe and effective in youth. Ongoing trial results and registry data will help define age-appropriate doses and titration schedules in children, clarify the ideal duration of therapy, and assess how durable weight loss is after cessation. In the SCALE Kids trial, daily liraglutide 3.0 mg produced a mean −5.8% BMI change (vs. +1.6% with placebo) over 56 weeks in 6–11-year-olds (35); similar studies of longer duration and of newer agents (e.g., semaglutide, once-weekly formulations) will determine whether even greater sustained weight loss can be achieved and maintained. Large-scale, long-term follow-up (including open-label extensions and real-world registries) is needed to monitor rebound weight gain after stopping therapy, as early reports suggest weight tends to increase once medication is discontinued (43). These longitudinal data will also reveal whether continuous therapy is required and how to taper or re-initiate treatment in growing children.

Another key research frontier is understanding how GLP-1 RAs affect growth, puberty, and psychosocial development. So far, trials have not demonstrated gross impairment of growth or bone mineral density, and some data suggest possible bone-protective effects, but pediatric data are limited (37). Because GLP-1 RAs reduce appetite and caloric intake, investigators must verify that children achieve adequate nutrition for normal linear growth and pubertal progression. Prospective studies should systematically measure height velocity, Tanner staging, and bone density in treated patients. In parallel, the impact on psychosocial health warrants urgent study. Rapid weight loss in youth may draw peer attention and could carry positive or negative emotional consequences. Early qualitative studies and longitudinal surveys will be needed to assess changes in self-esteem, body image, eating behaviors, and the risk of disordered eating. Côté and colleagues highlight the concern that GLP-1 RAs treatment *could* exacerbate social stigmatization or prompt feelings of “shame” if weight rebounds after stopping therapy (43). Research should also examine health equity: expanding medication use may reduce overall obesity rates but could widen racial/ethnic disparities if access remains unequal (43). Future trials and observational registries must therefore include diverse populations and evaluate socioeconomic and demographic predictors of treatment uptake and adherence.

Optimizing the real-world implementation of these therapies is another important avenue. Studies are needed on effective combination strategies (medication plus lifestyle or other drugs), and on the use of digital health tools to support adherence and healthy habits. For instance, integrating GLP-1 RA therapy with nutrition counseling, structured exercise programs, and behavioral therapy may enhance outcomes. Innovative trials could test whether mobile apps, telemedicine follow-up, or activity monitors improve long-term weight management with GLP-1 RAs (43). Finally, it will be essential to monitor for and understand rare or long-term adverse effects: dedicated pediatric safety registries (capturing renal function, growth parameters, and any emerging signals over years of use) will be essential. In sum, ongoing and future research will define the best pediatric dosing regimens, clarify which patients derive maximal benefit, and ensure that the long-term efficacy and safety of GLP-1 RAs in youth are fully understood.

## 4 Conclusion

In summary, GLP-1 RAs have become an essential option in the management of pediatric obesity. Recent trials indicate that these drugs may lead to significant weight loss and metabolic improvements in adolescents and younger children. When used under expert supervision and combined with lifestyle therapy, GLP-1 RAs generally show an acceptable safety profile in youth, primarily associated with mild gastrointestinal side effects. This review synthesizes the latest efficacy, safety, and guideline evidence, highlighting how GLP-1 RAs can be integrated into child and teen obesity treatment. We emphasize that clinicians should adopt a holistic approach—monitoring growth and mental health, continuing nutrition and activity counseling, and obtaining family support—when prescribing these agents. We propose a clinical medication management process that encompasses review, screening, combination therapy, education, monitoring, and follow-up.

At the policy level, our analysis points out the necessity of improving access and funding for pediatric obesity pharmacotherapy to avoid further widening disparities. Additionally, we have identified critical knowledge gaps that require future research, including the definition of long-term therapy outcomes, understanding impacts on development and psychosocial well-being, and determining optimal combination therapies. Accumulating data will guide evidence-based practice, enabling providers to use GLP-1 RAs safely and effectively for eligible youth. By remaining attentive to emerging evidence and addressing these research gaps, the pediatric community can better understand the potential role of GLP-1 receptor agonists in managing pediatric obesity.
